# A minimally invasive animal model of atherosclerosis and neointimal hyperplasia for translational research

**DOI:** 10.1186/s41747-025-00558-1

**Published:** 2025-02-06

**Authors:** Max L. A. Ebert, Vanessa F. Schmidt, Osman Öcal, Anne von Thaden, Olaf Dietrich, Bastian Popper, Sandra Elges, Max Seidensticker, Jens Ricke, Melanie A. Kimm, Astrid Jeibmann, Moritz Wildgruber

**Affiliations:** 1 Department of Radiology, University Hospital, LMU Munich, Munich, Germany; 2 Department of Diagnostic and Interventional Radiology, University Hospital Heidelberg, Heidelberg, Germany; 3 Veterinary Practice, Hohenpeißenberg, Germany; 4 Biomedical Center, Core Facility Animal Models, Faculty of Medicine, Ludwig-Maximilians-Universität München, Großhaderner Straße 9, 82152 Planegg-Martinsried, Germany; 5 Gerhard-Domagk-Institute of Pathology, Münster University Hospital, Münster, Germany; 6 Institute of Neuropathology, University Hospital Münster, Münster, Germany

**Keywords:** Endothelium (vascular), Models (animals), Neointima, Plaque (atherosclerotic), Stents

## Abstract

**Background:**

A variety of animal models has been developed for research on atherosclerosis and neointimal hyperplasia. While small animal models contain limits for translational research, we aimed to develop an atherosclerosis model with lumen-narrowing plaques to foster basic research in vascular biology, the development of new angioplasty devices, and vessel wall imaging approaches.

**Methods:**

Endothelial denudation was performed *via* a minimally invasive approach through the auricular artery, followed by stent-retriever mediated endothelial injury in New Zealand White rabbits (*n* = 10). Along with a high-fat diet, the rabbits developed lumen-narrowing atherosclerosis and neointimal hyperplasia of the iliac arteries within a 6-week period after mechanical injury. The stent-retriever method was compared with a conventional rabbit model (*n* = 10) using balloon denudation via surgical access, and both models were analyzed with a particular focus on animal welfare. Fisher’s exact, Mann–Whitney *U*, and unpaired *t*-tests were used.

**Results:**

The average time for the entire procedure was 62 min for the balloon group and 31 min for the stent-retriever group (*p* < 0.001). The stent-retriever model resulted in less periprocedural morbidity (including expenditure, intubation time, anesthetics, and end-tidal CO_2_ level) and mortality (40% mortality in the conventional group compared to 0% in the stent-retriever model, *p* = 0.011), while generating lumen-narrowing atherosclerotic lesions with key features as compared to humans as revealed by time-of-flight magnetic resonance imaging and histology.

**Conclusion:**

We developed a minimally invasive model of iliac atherosclerosis with high reproducibility and improved animal welfare for translational research.

**Relevance statement:**

This advanced rabbit model could allow for translational research in atherosclerosis, including pharmacological investigations as well as research on interventional angioplasty procedures.

**Key Points:**

- Rabbit models show similar lipid metabolism as humans.
- Stent-retriever mediated endothelial denudation causes neointimal hyperplasia and lumen narrowing.
- This minimal invasive model allows for clinical translation, including pharmacological investigations and vessel wall imaging.

**Graphical Abstract:**

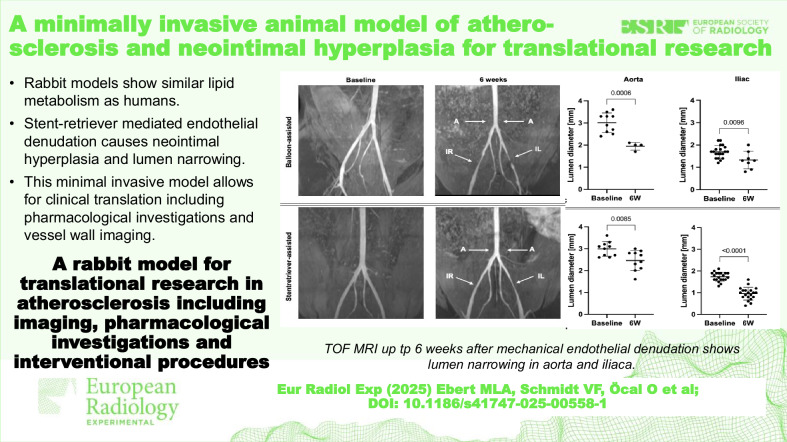

## Background

Atherosclerosis represents a chronic and progressive inflammatory condition resulting from multifactorial causes, leading to the gradual obstruction of arterial blood vessels over time. This predominantly affects the coronary, carotid, and peripheral arteries, causing either chronic impairment of blood flow or a sudden decrease in organ perfusion in case of acute vessel occlusion. The well-established standard to treat lumen-narrowing atherosclerotic plaques—percutaneous transluminal angioplasty—still shows a relapse rate of up to 50% within the first 12 months due to neointimal hyperplasia and subsequent restenosis [1–3]. Neointimal hyperplasia results from abnormal smooth muscle cells, which migrate from the tunica media towards the intimal layer and continuously proliferate in response to injury [4].

To develop targeted approaches minimizing neointimal hyperplasia, for example, *via* drug-eluting angioplasty devices, a profound understanding of the biological mechanisms of restenosis is required. Various animal models have been developed and are being used, from small rodents up to non-human primates [5]. High-throughput models of rats and mice lack translational potential due to manifold dissimilarities to humans. Most importantly, various injury models in conjunction with special diets result in intense vascular inflammation and some even in lumen narrowing stenosis [6]. Models in larger animals instead are cumbersome and expensive in terms of the technical procedure and animal housing. In this regard, rabbit models have been shown to be suitable for studying restenosis because of their size as well as cardiovascular and metabolic similarities to humans [7]. Atherosclerotic lesions are frequently generated by a combination of a high-fat diet (HFD) and mechanical injury of the endothelial cell layer [8, 9].

Previous rabbit models have proven to generate lumen narrowing atherosclerotic lesions and neointimal formation, but also come with several considerable disadvantages. The overstretching of the vessel wall by use of oversized catheters and balloons, as well as the substantially high level of procedural invasiveness, correlates with a certain risk of stroke and wound inflammation [10–13]. Alternative models in other larger animal models, such as dogs or non-human primates, have been described for similar purposes; however, the associated benefits have to be weighted critically against the increased maintenance efforts and costs, but even more importantly, ethical concerns when using those far developed species [5]. The rabbit, thereby, is considered a good compromise between high translational potential and reasonable maintenance and is ethically justifiable.

We aimed to establish a minimally invasive rabbit model of atherosclerosis that creates reproducible lumen-narrowing plaques without massive overstretching of the vessel wall, which regularly results in rupture up to the tunica media and increased vessel wall hemorrhage [5, 14, 15]. A model more closely resembling human pathophysiology was thought to enhance translatability, especially for endovascular therapy similar to clinical interventions for lumen-narrowing lesions, while simultaneously providing eased feasibility, improved animal welfare as well as a low rate of failure. The stent-retriever model is aimed to be well suited for both interventional research, such as the development of novel drug-eluting devices, antithrombotic therapies as well as gene therapies addressing neointimal hyperplasia, as well as to foster research on vessel imaging of atherosclerosis, such as imaging endothelial dysfunction, vascular wall inflammation, early plaque formation and response to angioplasty.

## Methods

### Experimental protocol

The ethical approval of the experiment was granted by the Government of Bavaria, Germany (Protocol Number: ROB-55.1-2532.Vet_02-19-178).

The experimental setting consisted of 20 male New Zealand White rabbits, 18 weeks of age, and 3–4 kg of weight at the beginning of the experiment. The animals were housed separately with about 2 m^2^ of space each and provided with enrichment items as well as hiding options and nesting material [16]. A 12-h day and night rhythm was maintained, including dusk and dawn.

The rabbits were fed an atherogenic high-fat and high-cholesterol diet (Diet-Nr. #290009, Altromin, Lage, Germany) to which they became accustomed within 4 weeks: 1st week 25% HFD, 2nd week 50% HFD, 3rd week 75% HFD, 4th week 100% HFD. The diet was complemented by the distribution of hay and water ad libitum. Control and care of well-being was provided daily while scoring of vital parameters and general health was executed at least twice a week. Surgical experiments were performed by a team of two interventional radiologists with > 10 years of experience in vascular procedures as well as with previous experience in rabbit research models, while the periprocedural care, as well as anesthesia, was conducted by a dedicated veterinarian experienced in animal research models.

The animals were split randomly into two groups of 10 rabbits each. One group to receive the conventional transcarotid balloon injury model, and the other one to receive the new minimally invasive trans-auricular stent-retriever induced arterial denudation model. To ensure optimal comparability, we used isoflurane narcosis sustained through an automatic ventilator system. Upon anesthesia induction (introduced by intravenous propofol), the following medical regimen was applied: for maintenance of analgesia Fentanyl (0.004 mg/kg intravenously every 30 min) and metamizole (60 mg/kg subcutaneously) were firstly injected. enrofloxacin 7.5 mg/kg was injected intravenously before vessel puncture or open surgery in order to prevent infections, while 0.07 mg/kg Aspirin and 45 IU/kg heparin were injected immediately before endothelial denudation to prevent acute thrombus formation. The ventilator mode was set on pressure control with 25–30 breaths/min and a basic isoflurane setting of 2.0 vol%, adjusted as needed. Digital pulse oximetry and capnography were used to monitor vital signs.

**Fig. 1:**
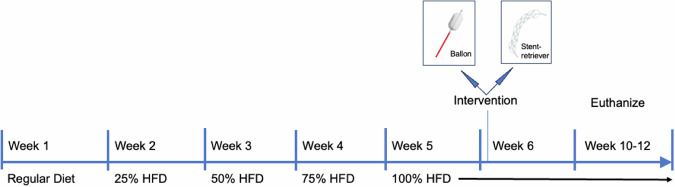
Experimental workflow. All animals were acclimatized to the HFD over a period of 4 weeks. Balloon-assisted intervention or stent-retriever-mediated endothelial denudation was performed in week 6. Animals were sacrificed at weeks 10–12, and arteries were histologically analyzed. HFD, High-fat diet

Following the intervention, rabbits in both groups were maintained for up to 6 weeks on the atherogenic diet until receiving magnetic resonance imaging (MRI) for assessment of lumen-narrowing lesions. Following MRI in deep sedation, animals were euthanized, and vessel samples of the aortic and iliac arteries were obtained. An overview of the experimental setup is shown in Fig. 1.

### Conventional transcarotid balloon injury technique

Following general anesthesia, the rabbit was positioned supinely, the throat area being meticulously shaved and disinfected with povidone [14, 17]. Access to the carotid artery was achieved through a 2–3 cm lateral incision along the trachea. Sutures with a 4-0 vicryl filament are placed upstream and downstream of the vascular access site. The exposed common carotid artery was punctured through the Seldinger technique with a micropuncture set using a 21-G needle and a 0.018-inch introducer wire (Micropuncture Pedal Introducer Access Set, Cook Medical, Bloomington, IN, USA), followed by insertion of a 5-F sheath (Radiofocus, Terumo Europe NV, Leuven, Belgium). Thereupon, guided by x-ray fluoroscopy after digital subtraction angiography using Imeron 300 M (Bracco, Germany) diluted 1:1 with normal saline, the iliac arteries were fathomed with a 0.018-inch guidewire. Subsequently, a 3-F Fogarty catheter (Edwards Life Sciences, Irvine, CA, USA) was positioned within the external iliac artery at the level of the iliac crest. After inflation, the catheter was drawn past the iliacal bifurcation towards the aorta. On average, the overstretching was more than twice: 5-mm inflated balloon diameter with a 3-F Fogarty catheter in comparison to ∼the 2-mm average iliac artery diameter in New Zealand White rabbits [5]. This procedure was repeated three times on each side, followed by a control angiogram to screen for possible dissection and acute vessel obstruction. Afterward, the accessed carotid artery was ligated, and the wound was sutured in a two-layered manner and shielded using an antiseptic aluminum spray.

### Transauricular stent-retriever injury technique

We modified the previously described approach in order to be able to conduct the model through minimally-invasive vascular access, obviating open surgery, and reducing (balloon-)catheter-induced overstretching of the vessel wall. As the stent-retrievers require only a 0.021-F microcatheter system, access *via* the central auricular artery is deemed feasible.

**Fig. 2:**
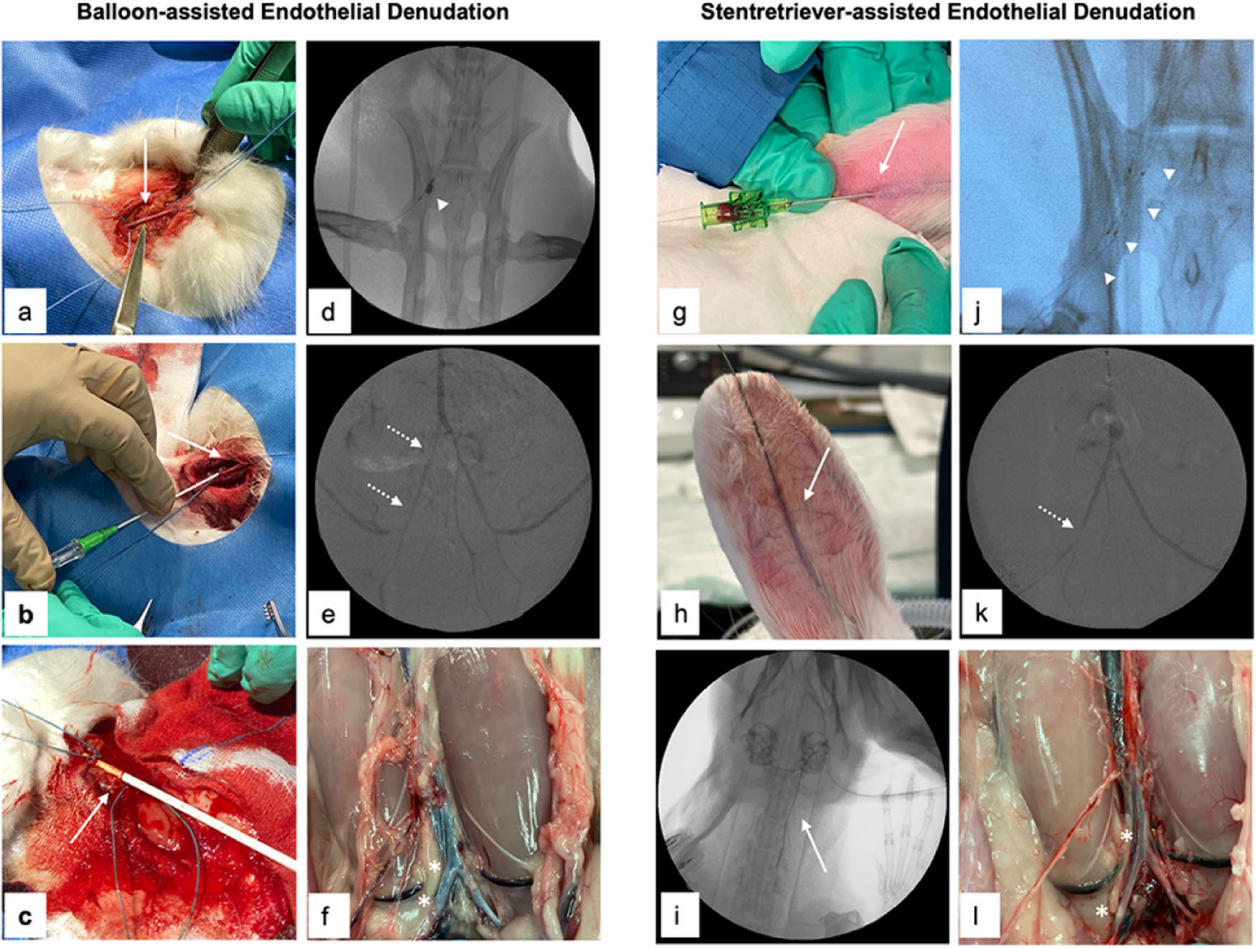
Comparison of balloon-assisted endothelial denudation *versus* stent-retriever-mediated endothelial denudation. For the conventional balloon-assisted endothelial denudation, the carotid artery is operatively exposed (**a**, arrow) and punctured with a 21-G needle (**b**, arrow), followed by insertion of 5-F sheath in Seldinger technique (**c**, arrow). After catheterization of the iliac artery the Fogarty balloon catheter device is placed within the external iliac artery, inflated (**d**, arrowhead), and pulled back into the distal aorta three times, followed by the same procedure on the contralateral side leading to local endothelial denudation and acute vasospasm (**e**, dotted arrows). Six weeks after the procedure the animals are euthanized, and macroscopic plaque formation is visible (**f**, asterisk). For the stent-retriever technique the *arteria auricularis caudalis* is punctured (**g**, arrow) with a 21-G needle and a 0.014-inch guidewire inserted into the artery (**h**, arrow) towards the common carotid artery (**i**, arrow) followed by the microcatheter. The stent-retriever is unfolded within the external iliac artery (**j**, arrowheads) and pulled backwards to the infrarenal aorta, leading to acute vasospasm as a result of the endothelial injury (**k**, dotted arrow). Six weeks later, macroscopically visible plaques are present along the aortic bifurcation and iliac arteries (**l**, asterisk)

For this approach, the rabbit was positioned in a prone stance. The area of access along the *ramus medius* of the *arteria auricularis caudalis* is shaved and prepared using a local anesthetic Xylocain-Prilocain ointment (EMLA, Aspen Germany, Munich, BY, Germany), promoting vessel dilatation. Subsequently, a modified Seldinger technique was executed: the artery was punctured percutaneously using a 21-G puncture needle, and a 0.014-inch guidewire (Fathom, Boston Scientific, Marlborough, MA, USA) was introduced. A 1-mm incision was crafted along the guidewire with precision scissors, enlarging the orifice to accommodate the microcatheter. The 0.014-inch guidewire was subsequently advanced under x-ray guidance towards the iliac arteries, followed by a 1.9-F microcatheter (Echelon 14 or Rebar 14, eV3 Neurovascular, Irvine, CA, USA). Next, the stent-retriever (Solitaire 4 mm × 20 mm, Medtronic, Minneapolis, MN, USA) was positioned, unfolded, and pulled backward past the aortic bifurcation. This procedure was equally repeated three times on each side. Following the final angiogram and subsequent catheter removal, hemostasis at the puncture site was obtained through manual compression for 2 min. The antiseptic aluminum spray was used to protect and cover the wound. The major steps for each technique are shown in Fig. 2.

### Postinterventional animal care

After the intervention, the animals were treated with analgesic (buprenorphine 0.025 mg/kg, metamizole 60 mg/kg) and low-dosed anticoagulant (enoxaparin 60 IU/kg) medication for at least 48 h, supplemented by additional doses if needed and with doses tapered down as clinically possible. The rabbit’s general medical condition, weight, and behavior were assessed meticulously for 2 days post intervention. The healing of wounds and hematomas as well as food and water intake were controlled daily for the following week.

For assessment of periprocedural and postprocedural animal welfare, morbidity, and mortality the following parameters were assessed: procedure time of the endothelial injury, time from end of intervention until extubation, minimum and maximum end-tidal carbon dioxide (etCO_2_) levels during the procedure, subjective post-procedure wake-up quality, overall procedure associated complications, post-interventional weight loss, food/water consumption, morbidity and mortality (Table 1).

**Table 1:** Procedural details, including procedure-related morbidity and mortality data

Perioperative characteristics	Stent-retriever (*n* = 10)	Balloon (*n* = 10)	*p*-value
Procedure time (min)	30.50 ± 8.6^a^	62.00 ± 13.8^a^	< 0.001^c^
Time from end of intervention until extubation (min)	2.60 (0)^b^	6.10 (8)^b^	0.076^d^
etCO_2_ level			
Minimum (mm Hg)	28.0 ± 2.6^a^	24.1 ± 2.2^a^	0.002^c^
Maximum (mm Hg)	31.5 (2.5)^b^	28.5 (7.25)^b^	0.049^d^
Wake-up quality			
Good	10/10 (100%)	1/10 (10%)	< 0.001^e^
Moderate	0/10 (0%)	8/10 (80%)	
Impaired	0/10 (0%)	1/10 (10%)	
Overall complications			
Hindlimb ischemia	0/10 (0%)	7/10 (70%)	0.011^e^
Sudden death	0/10 (0%)	3/10 (30%)	0.210^e^
Termination criteria fulfilled	0/10 (0%)	2/10 (20%)	0.473^e^
	0/10 (0%)	2/10 (20%)	0.473^e^
Postinterventional weight loss			
1 day after intervention (kg)	0.02 ± 0.06^b^	0.10 ± 0.07^b^	0.070^c^
2 day after intervention (kg)	0.04 ± 0.11^a^	0.01 ± 0.06^b^	0.195^c^
Postinterventional food/water consumption			
Good	10/10 (100%)	5/10 (50%)	0.033^e^
Impaired	0/10 (0%)	5/10 (50%)	
Mortality	0/10 (0%)	6/10 (60%)	0.011^e^
Lumen narrowing plaques	10/10 (100%)	4/4 (100%)	> 0.999^e^

^a^ Mean ± standard deviation

^b^ Median (interquartile range)

^c^ Unpaired *t*-test

^d^ Mann–Whitney *U*-test

^e^ Fisher exact test

### Magnetic resonance imaging of rabbit vasculature

MRI was performed on a 3.0-T whole body system (MAGNETOM Skyra, Siemens Healthineers, Erlangen, Germany) with a bore diameter of 70 cm. The gradient system had a maximum gradient strength of 45 mT/m and a slew rate of 200 T/m/s. A circularly polarized extremity coil (Siemens Healthineers) with a minimum inner dimension of 154 mm (total dimensions: 265 × 360 × 310 mm) was used as the receiver coil.

After deep anesthesia of the rabbit using 0.25 mg/kg medetomidine and 25 mg/kg ketamine intramuscular injection, the animals were inserted lengthwise as the upper coil part was removable. Thereupon, the rabbits were placed feet-forward-prone in the coil. The rabbits inside the coil were positioned in the isocenter of the *x*-*z* plane using the light visor. After scout scans, the applied MRI protocol was based on a time-of-flight magnetic resonance angiography (MRA) sequence (flip angle 17°; echo time 3.43 ms; repetition time 52 ms; bandwidth 185 Hz/pixel) with a field of view of 181 × 199 mm^2^ and an acquisition time of 348 s. Following the last MRI at 6 weeks after the intervention, the animals were euthanized in deep anesthesia (0.25 mg/kg medetomidine and 25 mg/kg ketamine, intramuscular injection) using 400 mg/kg intravenous pentobarbital and the infrarenal aorta as well as both iliac arteries (including the pelvic bifurcation) were harvested.

### Lumen diameter assessment of rabbit vasculature

For image analysis of the diameters of aortic and iliac vessels before and after endothelial injury, Visage Imaging software (Visage Imaging GmbH, Berlin, Germany) was used. The vessel diameters were measured using the time-of-flight MRA sequence images at three predefined positions perpendicular to the vessel axis (distal aorta immediately before the aortic bifurcation and the external iliac artery 2 cm distal of the iliac artery bifurcation). All assessments were performed similarly in the first MRI scan series before endothelial injury and in the last MRI scan series (before euthanasia). The assessment was performed by two independent readers (diagnostic radiologists with > 5 years of experience in vascular MRI each) blinded to the groups and *ex vivo* analysis.

### Histology and immunohistochemistry

Tissues were formalin-fixed, paraffin-embedded, and stained with MSB-Lendrum staining (#12076.00100) (Morphisto GmbH, Offenbach am Main, Germany) according to standard protocols. For immunohistochemistry, a biotin-streptavidin peroxidase technique (DetectionLine PolyLink-HRP-Kit, DCS, Hamburg, Germany) was used. Sections were pretreated with citrate buffer (pH 6), target retrieval solution (pH 6.1; both Agilent Dako, Santa Clara, USA). The following primary antibodies were used: mouse anti-alpha-smooth muscle actin 1A4 (Thermo Fisher Scientific, Waltham, USA, #MA5-11547, 1:800, no pretreatment), mouse anti-CD31 (Novus Biologicals, Centennial, USA, NBP2-44342, 1:100, pH 6.1) and with mouse anti-MHCII/HLA DR + DP + DQ antibody (CR3/43) (Agilent DAKO, Santa Clara, USA, ab7856; 1:100; pH 6.0). All sections were scanned using NanoZoomer S360 MD (Hamamatsu, Hamamatsu City, Japan), and intimal thickening was analyzed using Aperio ImageScope-Pathology Slide Viewing Software (Version 12.3.3, Leica Biosystems, Nußloch, Germany).

### Statistical analysis

The Shapiro–Wilk test was used for the assessment of normality. Data are presented as means ± standard deviation in case of normal distribution or as medians and interquartile range (IQR) for skewed distribution. Descriptive statistics were used to analyze the distribution of rabbits among the different categories. Subanalyses between both rabbit groups were performed using Fisher’s exact test for categorical data and the Mann–Whitney *U*-test and unpaired *t*-test for metric data. Statistical testing was conducted using SPSS (Version 26.0, IBM Corp., Armonk, N, USA) and GraphPad Prism (Version 10.2.0, GraphPad Software, Boston, MA, USA) with *p* < 0.05 considered significant. All *p*-values were two-tailed.

## Results

### Interventional procedures

Ten animals underwent the conventional transcarotid balloon denudation, and ten underwent the novel transauricular stent-retriever mediated denudation. Three complete endothelial denudation passages were performed on each iliac artery in each rabbit of both groups.

Six rabbits were lost after the intervention: three rabbits had to be euthanized due to hindlimb ischemia (two on the day of surgery and one animal 1-day post-surgery), one rabbit had to be euthanized 1 day after the procedure due to lethargy, and heavy pain despite analgesia, and two rabbits died due to uncharted reasons (sudden death) at day 12 and 13 respectively, after endothelial denudation. All animal losses occurred in the balloon group. In two animals of the stent-retriever group, initial attempts to access the auricular artery failed, requiring a second intervention which was successful. In the stent-retriever group, all animals (*n* = 10) developed intimal hyperplasia with a 100% survival rate (Table 1). Even though all animals in the balloon group also developed atherosclerotic plaques and a neointimal hyperplasia, the mortality rate in the balloon group was 40%, which was significantly higher compared to the stent-retriever group (*p* = 0.011) (Table 1).

The average time needed for the entire procedure was 62 min for the balloon group and 31 min for the stent-retriever group (*p* < 0.001) (Table 1). The duration from the end of intervention until extubation showed a tendency to be faster in the stent-retriever group (*p* = 0.076). Furthermore, the awakening process demonstrated significantly improved outcomes within the stent-retriever group (*p* < 0.001) (Table 1). In addition, the shorter intervention time in the stent-retriever group also correlated with a lower intraoperative fentanyl usage (median 0.03 mg, IQR 0.02 mg) per animal in the balloon group *versus* 0.04 mg (IQR 0.02) per animal in the stent-retriever group (*p* = 0.067). Seven rabbits of the balloon group showed transient reflex bradycardia during critical parts of the surgery suggesting spikes in circulatory stress. The etCO_2_ levels differed significantly between the stent-retriever (minimum 28 mmHg ± 2.6 mm Hg; maximum 31.5 mm Hg, IQR 2.5 mm Hg) and the balloon group (minimum 24.1 mm Hg ± 2.07 mm Hg; maximum 28.5 mm Hg, IQR 7.25 mm Hg) (*p*_min_ = 0.002; *p*_max_ = 0.049) (Table 1). This data reflects a more stable expiratory etCO_2_ level without major fluctuations. Six animals of the balloon group and one of the stent-retriever group needed an over 30% increase in isoflurane dose during the intervention. Subjective wake-up quality was significantly better in the stent-retriever *versus* the balloon group (*p* < 0.001).

None of the 14 rabbits developed wound infections, necrosis, or similar complications related to the vascular access site during the observation time, and all hematomas and scars healed appropriately. Five animals in the balloon group showed impaired food and water intake after the intervention, none in the stent-retriever group (*p* = 0.033, Table 1). Although overall weight progression displayed no significant disparity between the two groups, a more pronounced post-procedural weight-loss was seen 24 h post-intervention in the balloon group (*p* = 0.070. Postinterventional buprenorphine demand was significantly lower in the stent-retriever group than in the balloon group (0.19 ± 0.09 mg *versus* 0.32 ± 0.15 mg, *p* = 0.036).

In summary, morbidity and mortality were substantially decreased by applying the minimally invasive stent-retriever technique.

### Development of lumen-narrowing stenosis

**Fig. 3:**
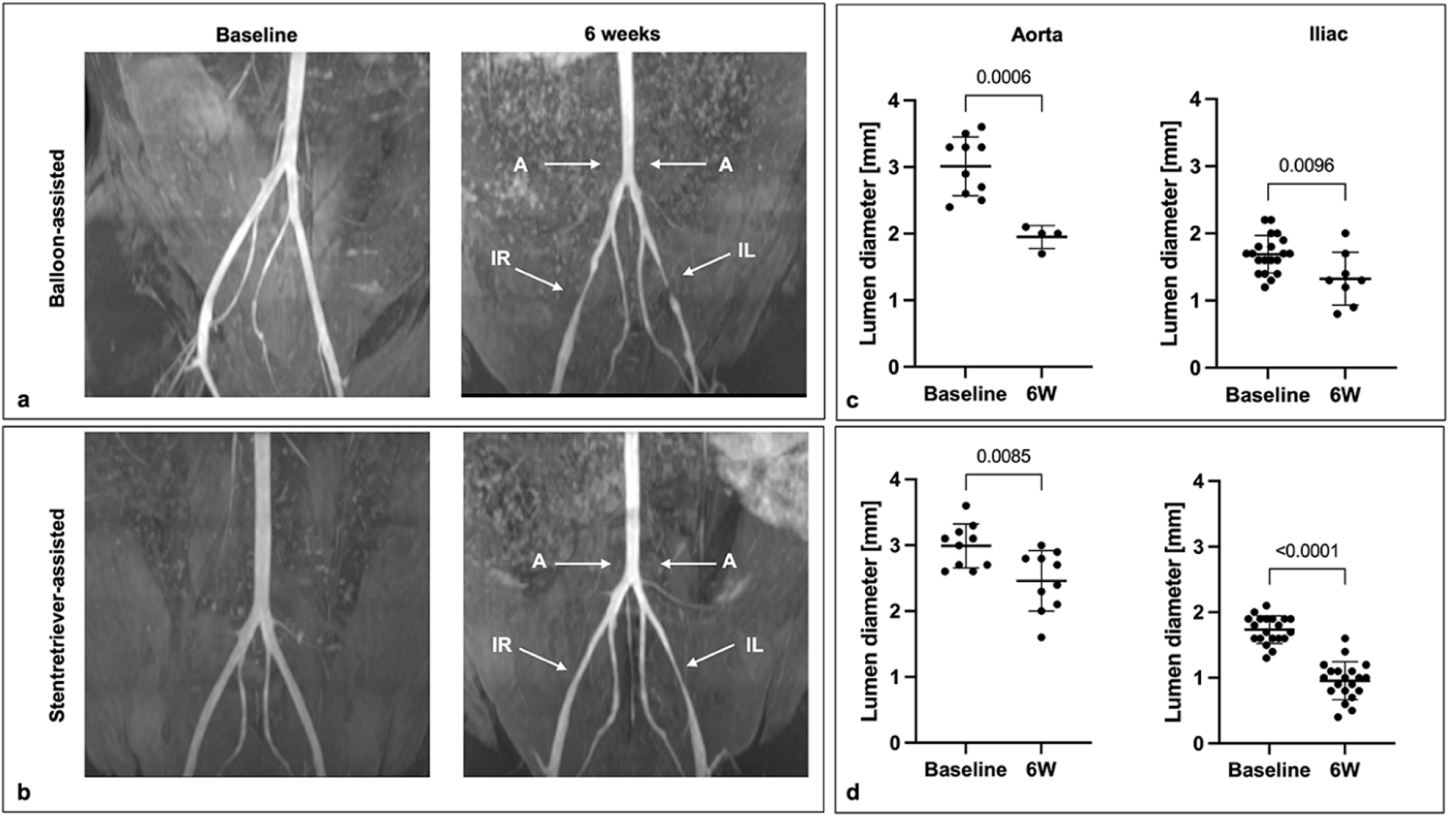
Assessment of lumen-narrowing atherosclerosis. **a**, **b** Time-of-flight magnetic resonance angiography was performed 6 weeks after mechanical injury in both groups revealing lumen-narrowing lesions along the aortic bifurcation and iliac arteries. Vessel segments used for subsequent diameter assessment are marked with arrows. **c**, **d** Lumen diameter assessment revealed a significant reduction at 4–6 weeks compared to baseline both at the aortic bifurcation as well as the iliac artery

Time-of-flight MRA revealed lumen narrowing stenosis in the iliac arteries in both groups at 6 weeks post endothelial denudation (Fig. 3). The mean perfused lumen decreased significantly both at the aortic bifurcation from 3.01 mm to 1.95 mm (*p* < 0.001), as well as from 1.69 mm to 1.33 mm (*p* = 0.010) at the external iliac in the balloon group. The lumen decrease following endothelial injury was similarly significant in the stent-retriever group, at the aortic bifurcation from 2.99 mm to 2.46 mm (*p* = 0.009), and along the external iliac artery from 1.74 mm to 0.96 mm (*p* < 0.001). The luminal decrease was at least 10% of the original diameter in each group.

### Atherosclerotic lesion characterization

**Fig. 4:**
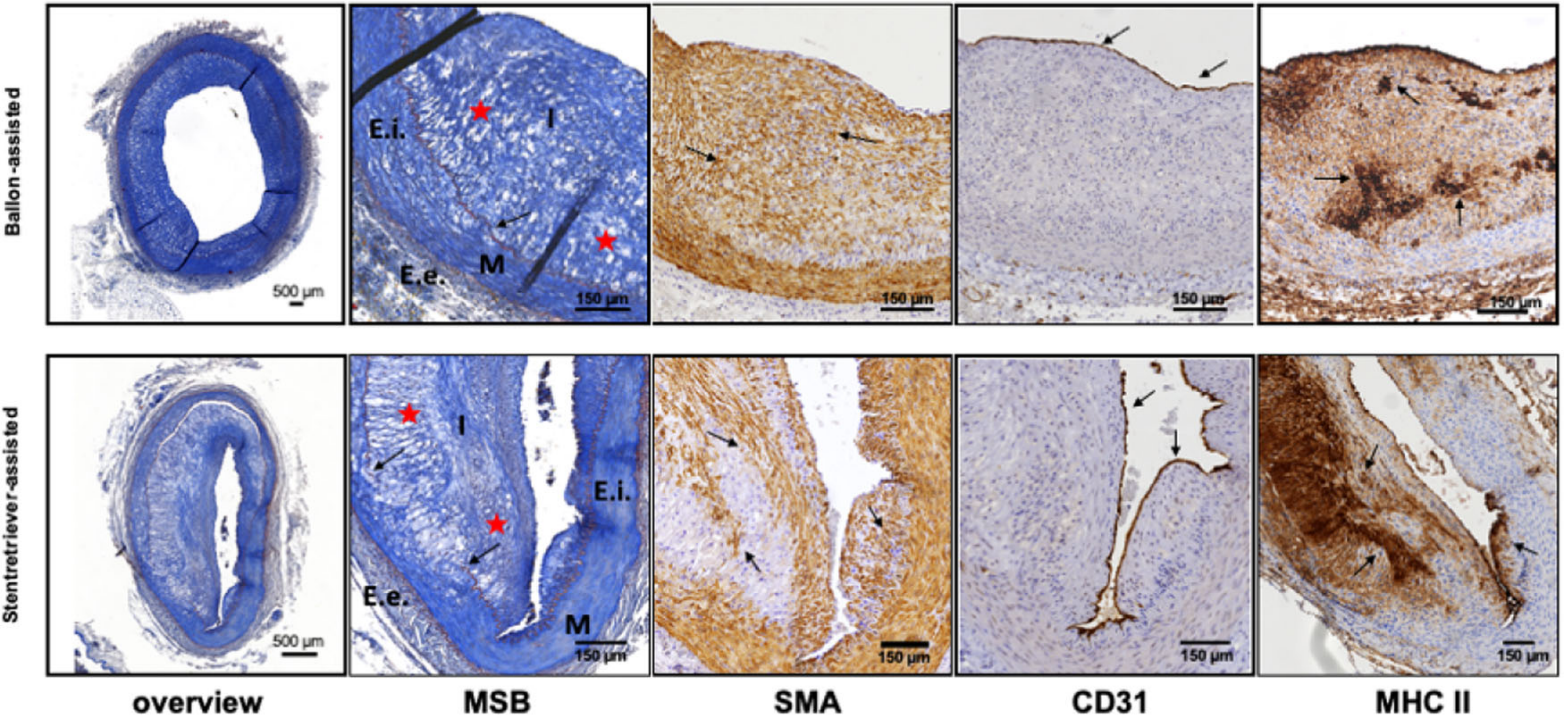
Histological assessment of atherosclerotic lesions. Staining of iliac vessel segments by MSB, SMA, CD31, and MHC II revealed characteristic features of inflammatory atherosclerotic lesions with the presence of macrophages, smooth muscle cell proliferation and migration towards the intima, disruption of the regular arterial wall architecture and an endothelium-covered hypertrophic intima. MSB, Martius scarlet blue; SMA, Smooth muscle actin; CD31, Cluster of differentiation 31; MHC II, Major histocompatibility complex class II

The development of atherosclerosis and lumen narrowing was finally investigated in a qualitative manner in both models post-mortem by assessing the major characteristics of neointimal hyperplasia [3] by histology/immunohistochemistry at 6 weeks post endothelial injury and 10 weeks after initiating the HFD (Fig. 4). Intimal thickening was detectable in all animals and the intima-to-media ratio did not significantly differ between the two different models applied (balloon group: 0.89 mm, stent-retriever group: 1.46 mm, *p* = 0.122). Both the *lamina elastica interna* and *externa* were disrupted to some degree in all animals and in addition, the arrangement of the cells in the intima was not as homogeneous as usually seen in the media. A loose extracellular matrix with collagen and fat deposits and proliferation, as well as infiltration of smooth muscle cells from the media into the intima, was detectable along both groups. Macrophages, visualized by Major Histocompatibility Complex class II staining, accumulated predominantly in the neointima, representative of the chronic inflammatory process. Furthermore, 6 weeks after endothelial injury, we detected a predominantly continuous endothelial barrier, shown by positive CD31 staining, in all animals of both groups. In summary, the balloon-mediated and the stent-retriever-mediated endothelial injury led to neointimal hyperplasia.

## Discussion

We have reported the successful establishment of a new, minimally invasive animal model for translational atherosclerosis research in rabbits.

Rabbit-based models have emerged as a good compromise between small rodents and large animals [5], allowing a reasonable throughput while at the same time providing large enough vascular anatomy allowing the investigation of interventional devices such as drug-coated balloons and stents [18–20]. The New Zealand White rabbit is large enough to permit detection of specific aspects of atherosclerosis and restenosis formation *via* translational molecular imaging approaches [21, 22]. One major advantage of rabbits compared to rodents is their lipid metabolism being similar to humans. With an intrinsically high cholesterol ester transfer protein−CETP activity, like humans, they transport most cholesterol via low-density lipoprotein and very low-density lipoprotein, which rodents do not [23]. Moreover, the rabbit apolipoprotein B, as well as the receptor for low- and very low-density lipoprotein, both in the liver and on the surface of macrophages, are similar to the human isoform [24, 25]. HFD combined with subsequent mechanical endothelial injury, therefore, offers the possibility to investigate pathologic events such as neointimal hyperplasia in a translationally meaningful way. While the cholesterol levels fed to rodents for proper plaque formation exceed by far the physiological cholesterol uptake in humans, 0.3–0.5% cholesterol has been shown to generate atheromas similar to human plaque formation in rabbits [14, 26]. As the lipid metabolism needed for sufficient atheroma formation resembles the human situation, one drawback of the most frequently used rabbit model was the severity of the vascular injury [14, 18–20]. As rabbit iliac arteries measure 2 mm in maximum diameter, the balloon denudation with a 4–5 mm Fogarty balloon device results in overstretching of the vessel wall, not resembling a human angioplasty procedure where over-dilatation is being avoided in order to reduce vascular damage.

We, therefore, aimed to further improve the translatability of the rabbit model and reduce the severity of endothelial injury to the vessel wall. Our hypothesis was that endothelial denudation of the iliac arteries can be performed with a stent-retriever device, usually used for mechanical thrombectomy in stroke treatment [27]. Stent retrievers, especially when being pulled repetitively across the vessel, injure the endothelium, without rupture of the internal elastic lamina. As the shear stress to the vascular wall is much less compared to conventional stents, and similarly, the radial force exerted onto the vessel wall is reduced compared to inflating a semi-compliant balloon to twice the actual vessel lumen diameter, as is frequently used to cause endothelial denudation [14, 28]. The effect of stent-retrievers, causing a moderate injury to the endothelium while avoiding massive overstretching with rupture of the remaining components of the arterial wall, is exactly the degree of injury needed to induce atherosclerosis in a translational manner. We postulated that utilizing a stent-retriever leads to sufficient endothelial denudation but, at the same time, avoids massive overstretching of the vessel compared to the conventional balloon model. The auricular artery access in the rabbit has been described before [29–31] and was recently described to gain access to the cerebral vasculature for stroke research [32], yet it has not yet been investigated for generating peripheral atherosclerosis. However, it comes with a quick learning curve and can be easily performed by a skilled interventionist. Moreover, there is no specific mechanical closure required to deal with the arterial access site, as it is required when accessing the carotid or iliac artery. The newly established technique allows performing sufficient endothelial injury for proper and reproducible plaque formation at a shorter procedure time and learning curve while simultaneously being associated with a decreased morbidity, mortality, and loss of research animals.

Concerning animal welfare, our approach indicated a significantly increased well-being, providing decreased pain and stress, both during and post-intervention. Additionally, the decreased need for analgesics, which limit the appetite of the animals and thereby impair regular food intake, was similarly considered beneficial. Gastrointestinal stasis, one of the most common causes of death in model rabbits, is nearly eradicated [33, 34]. To assess lumen-narrowing stenosis in rabbit iliac arteries time-of-flight MRA was considered most appropriate as it allows image reconstruction perpendicular to the vessel wall, at the same time avoiding the need for intravascular contrast agents.

The first limitation of this study is the lack of quantitative comparisons of key histopathological features between both groups, although plaque formation appeared similar when comparing the results from both groups qualitatively. This would allow judging potential differences with respect to plaque biology, vessel wall inflammation, and the degree and kinetics of neointimal hyperplasia more profoundly. As histology cannot be considered a quantitative approach, key features of plaque formation, such as dedicated metabolites in the vessel wall or deposition of certain radiological contrast agents could be compared by means of mass spectrometry imaging in the future [35, 36].

Another limitation is the lack of long-term data on plaque formation in comparison between the two models. While at 6 weeks, the lumen narrowing was more pronounced following balloon denudation, it is unclear if this was a result of the more intense injury and, similarly, if this difference holds true over a longer observation period. Now, with the new model being established, it needs to be further validated with respect to long-term differences in the vascular wall and forming atheromas. Additionally, the model needs to be further evaluated with respect to pharmacological interventions targeting atherogenesis. Various approaches aiming at restoring endothelial function, reducing vascular wall inflammation, and stabilizing ulcer-prone atheromas are under investigation [37–40], yet frequently limited to small animal models, which typically lack features like lumen-narrowing lesions as well as ulcerating plaques.

The modified rabbit model is thought to foster both research in interventional as well as diagnostic imaging. It will provide a new platform to study the effect of novel drug-eluting devices on vascular inflammation, neointimal proliferation and hyperplasia, and subsequent restenosis formation. Similarly, additive therapies, such as the development of novel antithrombotics are expected to continue to pose a challenge in vascular medicine, requiring suitable, medium to large-scale animal models [41]. Also, novel approaches of gene therapy targeting central gene hubs regulating pivotal atherogenic as well as atheroprotective signaling need to be studied extensively in animal models before pilot trials can be performed in humans [42].

With respect to diagnostic imaging, vascular radiology has long moved beyond imaging of vessel diameter and stenotic lesions. Especially sophisticated MRI techniques are now able to visualize endothelial permeability using macromolecular contrast agents [43], vascular inflammation using superparamagnetic iron-oxide-based nanoparticles targeting monocytes/macrophages [44], or to address specific aspects of neointimal hyperplasia using targeted probes and tracers [45]. The development of those novel imaging strategies requires profound validation, especially of the molecular and cellular targets. Similarly, for translational purposes, those imaging approaches are favorably developed in larger animal models that do not require dedicated ultrahigh-field MRI scanners.

In summary, we presented a novel approach for minimally invasive endothelial denudation in hypercholesterolemic rabbits, leading to human-like atherosclerotic lesions with lumen-narrowing stenosis. The model is fast, easy to perform, and comes with reduced animal loss. At the same time, this model allows for translational research in atherosclerosis, including pharmacological investigations such as restoring endothelial dysfunction, ameliorating vessel wall inflammation, and reducing the formation of rupture-prone atheromas, as well as research on drug-eluting devices. Moreover, gene therapies trying to impede both de novo atherosclerosis as well as neointimal hyperplasia are on the horizon. They need to be evaluated in translational animal models prior to trials on humans.

## Data Availability

The data that support the findings of this study are available from the MW on reasonable request.

## Abbreviations

etCO_2_: End-tidal carbon dioxide
HFD: High-fat diet
IQR: Interquartile range
MRA: Magnetic resonance angiography
MRI: Magnetic resonance imaging
